# Supplementary material for: Shear Stress and Sub-Femtomolar Levels of Ligand Synergize to Activate ALK1 Signaling in Endothelial Cells
Source: Cells. 2024 Feb 5;13(3):285. doi: 10.3390/cells13030285 (PMC10854672; doi:10.3390/cells13030285)
Supplement: Supplementary file 1 [file cells-13-00285-s001.zip › cells-2710015-supplementary.pdf]

cells MDPI

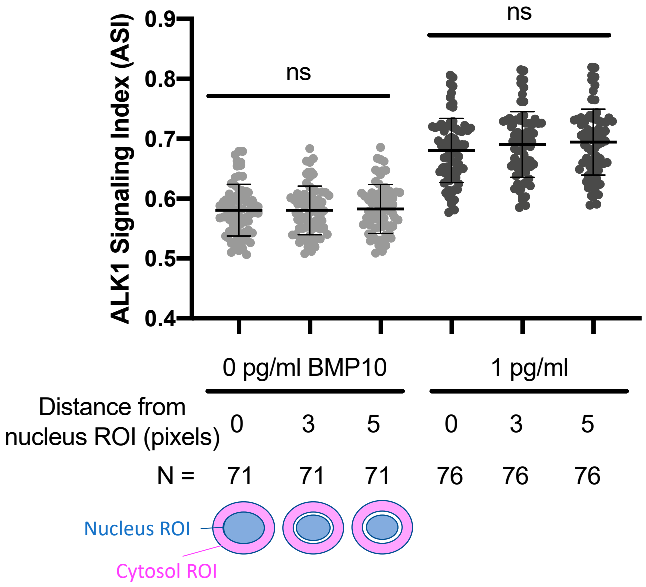

Supplementary Figure S1 (Supports Figure 1B). Validation of cytosolic region of interest (ROI) used to calculate ALK signaling index (ASI). Confluent HUVECs were serum starved for 4 hours in 0.2% BSA medium, treated for 45 minutes with 0 or 1 pg/mL BMP10, then fixed and stained for nucleus (DAPI) and pSMAD1/5/9. To calculate the ASI, the cytosolic ROI was drawn 0, 3, or 5 pixels (0, 1.3, or 2.2 μm) away from nuclear ROI, and resulting ASIs compared by one-way ANOVA with Tukey multiple comparisons test. ns = not significant. N = 3 independent experiments. Each dot represents 1 EC.

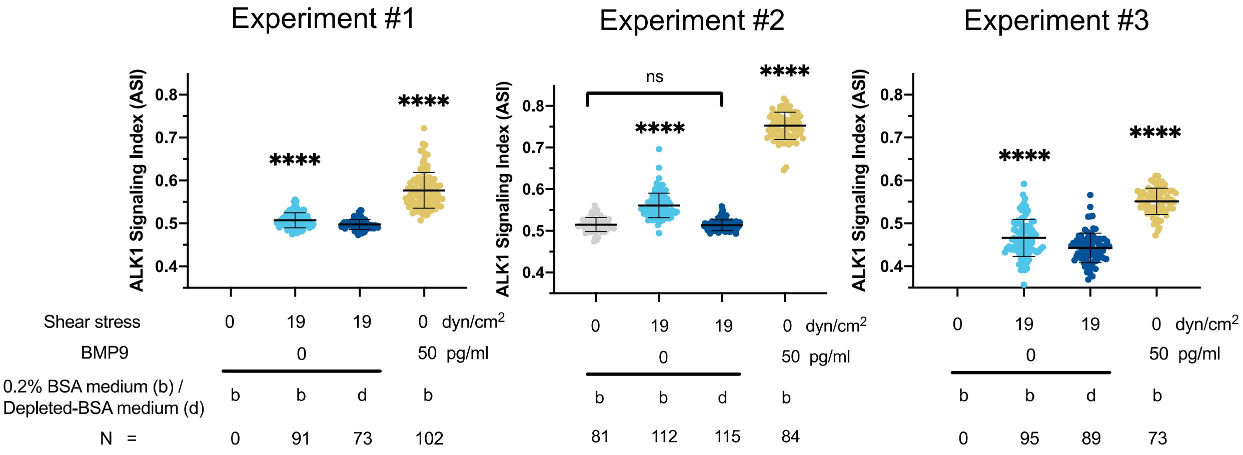

Supplementary Figure S2 (Supports Figure 3B). The effect of pSMAD1/5/9 localization between the ALK1-Fc depleted BSA medium and normal BSA medium. In all three trials, statistically significant differences are observed in pSMAD1/5/9 localization between the ALK1-Fc depleted BSA medium and normal BSA medium.

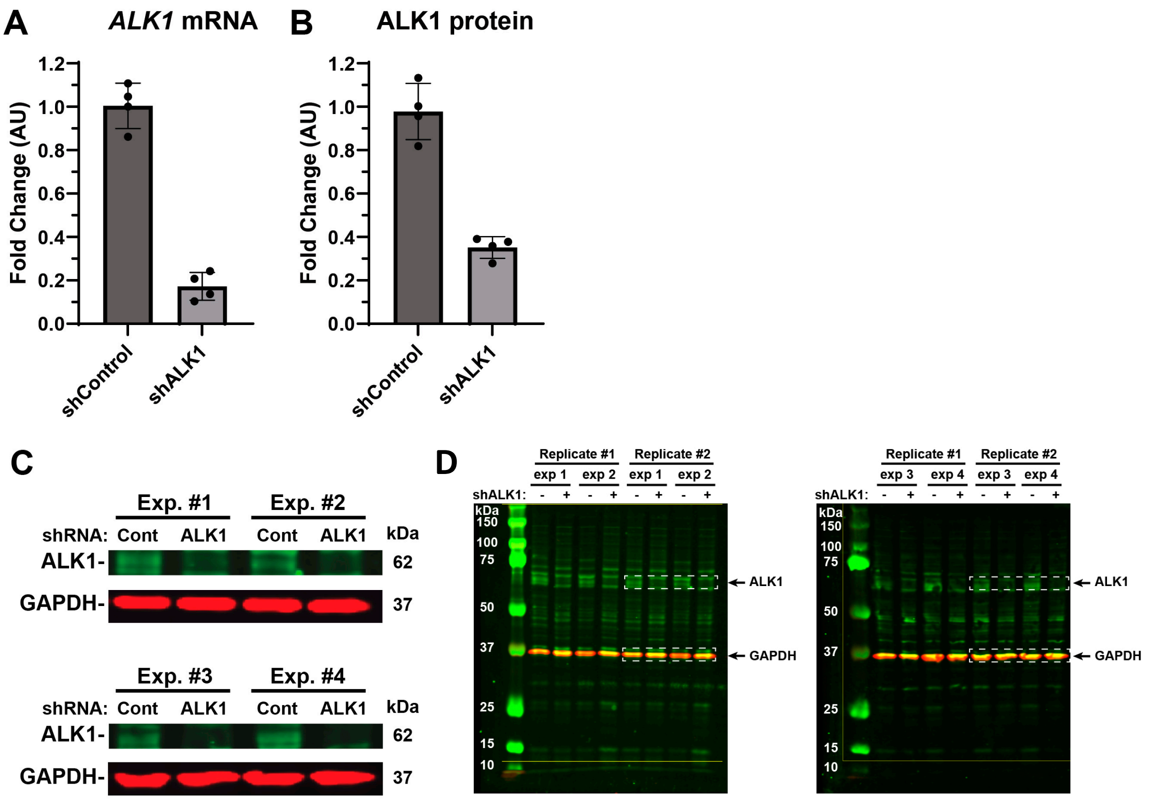

Supplementary Figure S3 (Supports Figure 4B). Knockdown efficiency of ALK1 in HUVECs. HUVECs were transduced with lentivirus carrying control or ALK1-targeted shRNA and analyzed at 6-days post-transduction for *ALK1* mRNA by RT-qPCR (**A**) and ALK1 protein by western blot, normalized to GAPDH (**B**,**C**). N = 3 independent experiments, with duplicate wells (as in **C**) averaged for each N.

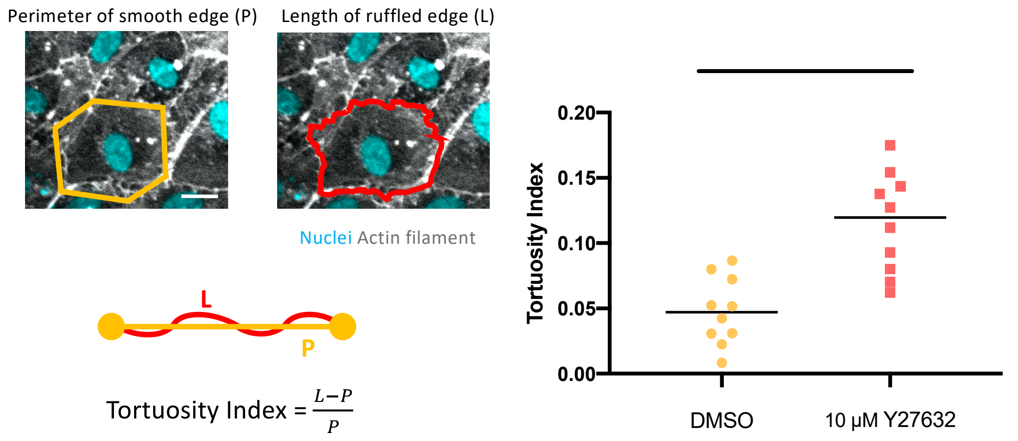

Supplementary Figure S4 (Supports Figure 5B). Y27632 enhances tortuosity of cell-cell junctions. Confluent HUVECs treated for 1 hour 45 minutes with 0.1% DMSO or 10 μM Y27632 in 0.1% DMSO. Nuclei were labeled with DAPI and the F-actin cytoskeleton was labeled with phalloidin, and the tortuosity index was calculated as indicated. N = 3 independent experiments. Each dot represents 1 EC. Two-tailed unpaired student's t-test. *** p =0.0002 . Scale bar = 20 μm.

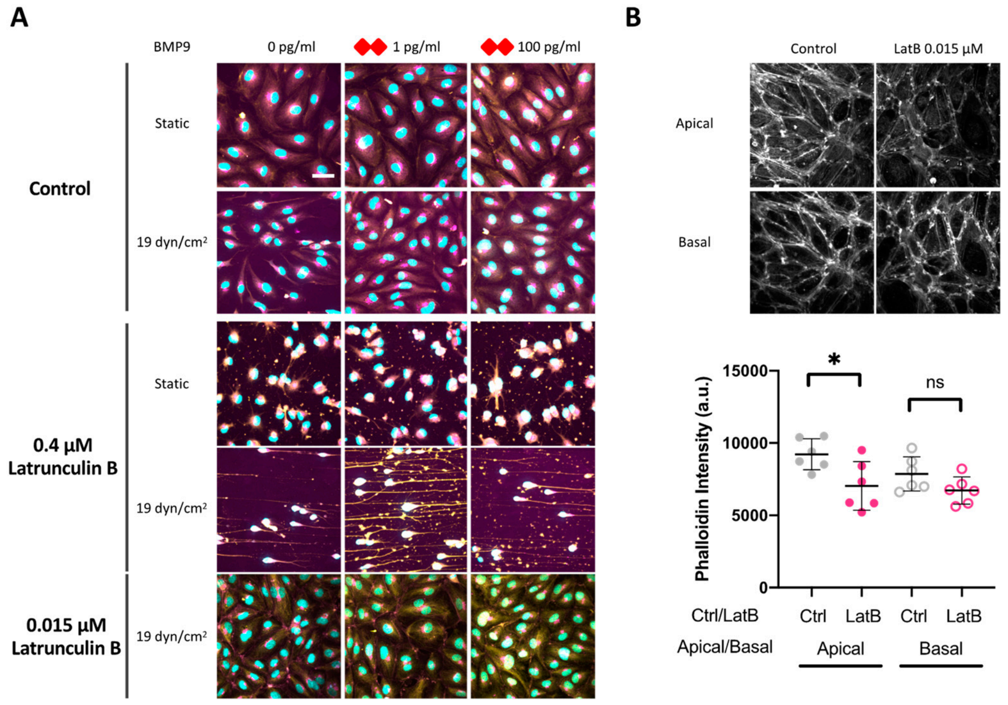

Supplementary Figure S5 (Supports Figure 5C). HUVECs treated with 0.015 μM Latrunculin B maintain normal EC morphology under shear stress. (A) Confluent HUVECs were grown in static conditions or under SS (19 dyn/cm^2) and treated with "standard" (0.4 μM) or low (0.015 μM) concentration of latrunculin B, or 0.4% DMSO for 75 minutes**.** Fixed cells were stained for the nucleus (cyan), pSMAD1/5/9 (yellow), and the Golgi (magenta). (B) Apical actin filament level was decreased with 0.015 μM Latrunculin B treatment, while basal actin remained unchanged under static condition. (stained by Acti-stain 670 phalloidin). Two-tailed unpaired student's t-test, * p = 0.0228. Scale bar = 40 μm.

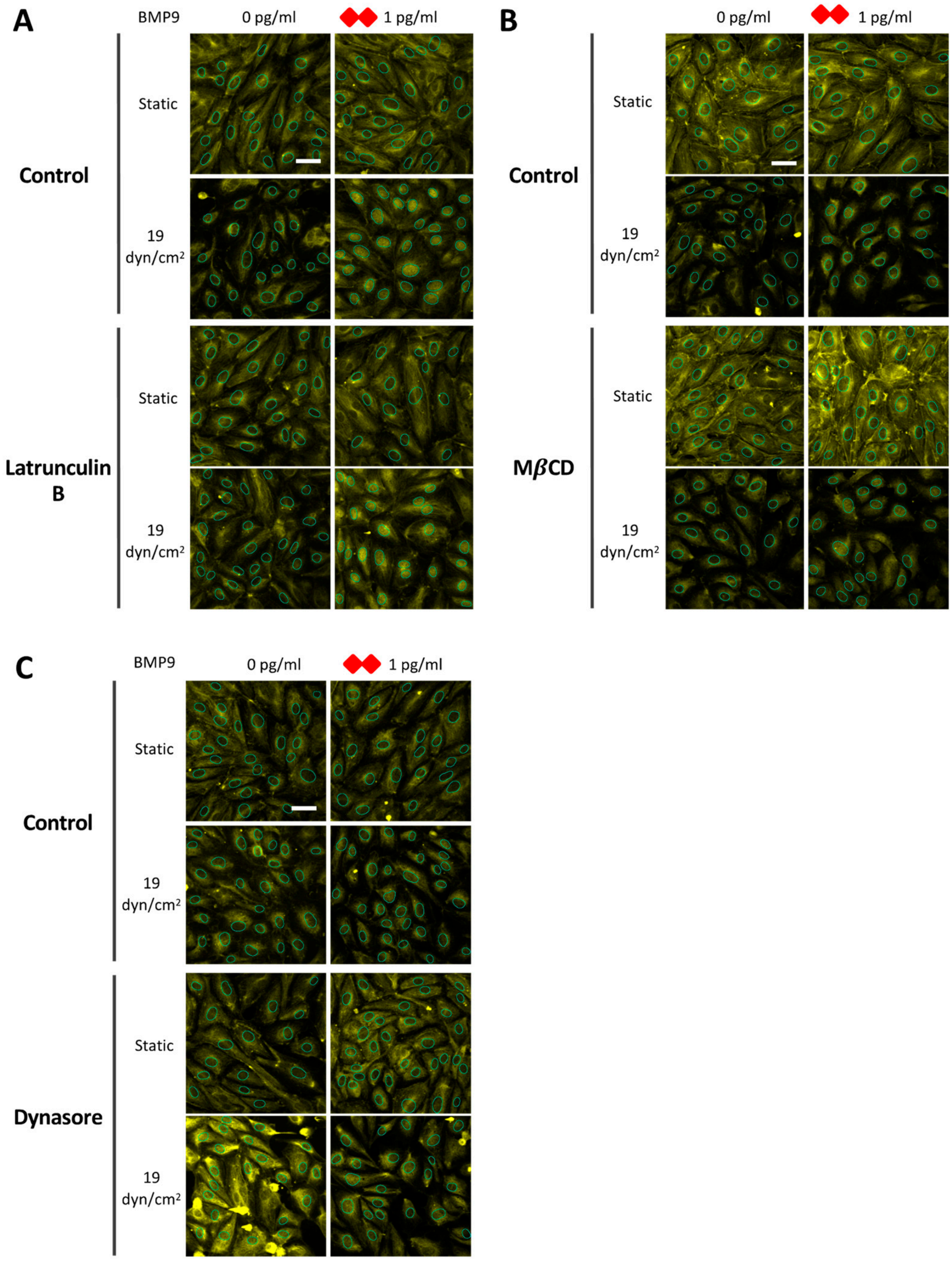

Supplementary Figure S6 (Supports Figures 5C, 5D, and 5E). Nuclear outlines (cyan) and the localization of pSMAD1/5/9 (yellow) in HUVECs treated with latrunculin B (A), MβCD (B) and Dynasore (C). The outlines of nuclei were segmented from the DAPI fluorescence. Scale bar = 40 μm.

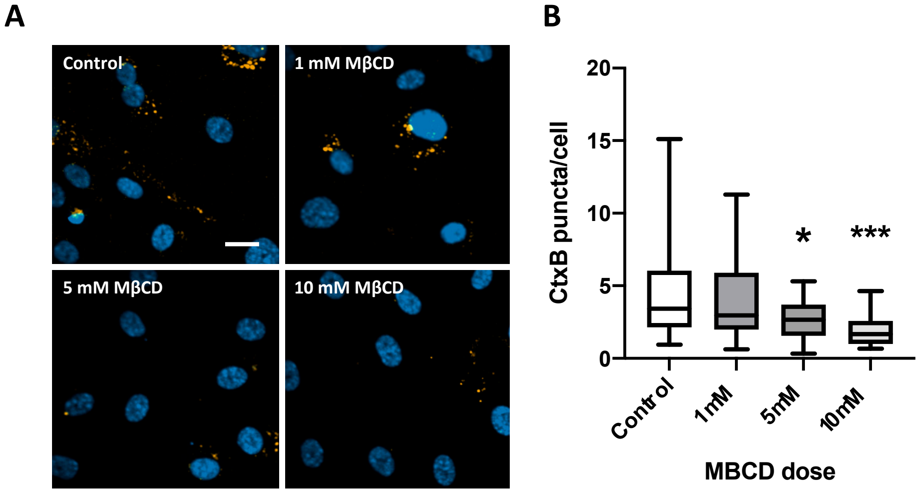

Supplementary Figure S7 (Supports Figure 5D). MβCD disrupts cholesterol-enriched microdomains. HUVECs were treated for 75 minutes with indicated concentrations of MβCD. Fixed cells were stain for nuclei (DAPI) and Cholera toxin subunit B (CtxB), and CtxB puncta counted in 4 fields and N > 250 cells per condition over 3 independent experiments. One-way ANOVA and Tukey post-hoc test, *p = 0.0217; ***p = 0.0005. Scale bar = 20 μm.

Supplementary Table S1 (Supports Figure 5). Phospho-SMAD1/5/9 mean intensities and standard deviation (SD) under static or flow condition.

BMP9 Conc. (pg/ml)	Treatment	Shear Stress (dyn/cm^2)	Mean	SD
0	Confluent	0	0.466	0.026
		19	0.489	0.041
	Sub-confluent	0	0.458	0.032
		19	0.534	0.036
1	Confluent	0	0.465	0.027
		19	0.571	0.051
	Sub-confluent	0	0.463	0.027
		19	0.567	0.046
0	-	0	0.509	0.014
		19	0.572	0.045
	Y27632	0	0.510	0.010
		19	0.572	0.046
1	-	0	0.514	0.016
		19	0.742	0.062
	Y27632	0	0.531	0.036
		19	0.752	0.063
0	-	0	0.451	0.030
		19	0.474	0.045
	Latrunculin B	0	0.451	0.032
		19	0.496	0.035
1	-	0	0.451	0.033
		19	0.603	0.046
	Latrunculin B	0	0.456	0.029
		19	0.607	0.040
0	-	0.425	0.028	0.425
		0.454	0.040	0.454
	MβCD	0.441	0.035	0.441
		0.452	0.033	0.452
1	-	0.430	0.027	0.430
		0.469	0.051	0.469
	MβCD	0.431	0.030	0.431
		0.505	0.049	0.505
0	-	0	0.446	0.031
		19	0.479	0.034
	Dynasore	0	0.435	0.029
		19	0.461	0.034
1	-	0	0.452	0.029
		19	0.561	0.069
	Dynasore	0	0.438	0.027
		19	0.552	0.059
